# An Aggregation-Induced Emission-Based Indirect Competitive Immunoassay for Fluorescence “Turn-On” Detection of Drug Residues in Foodstuffs

**DOI:** 10.3389/fchem.2019.00228

**Published:** 2019-04-24

**Authors:** Wenbo Yu, Ying Li, Bing Xie, Mingfang Ma, Chaochao Chen, Chenglong Li, Xuezhi Yu, Zhanhui Wang, Kai Wen, Ben Zhong Tang, Jianzhong Shen

**Affiliations:** ^1^Beijing Key Laboratory of Detection Technology for Animal-Derived Food Safety, Beijing Laboratory for Food Quality and Safety, Beijing Advanced Innovation Center for Food Nutrition and Human Health, College of Veterinary Medicine, China Agricultural University, Beijing, China; ^2^Division of Life Science, Department of Chemical and Biological Engineering, Department of Chemistry, Hong Kong Branch of Chinese National Engineering Research Center for Tissue Restoration and Reconstruction, Institute for Advanced Study, The Hong Kong University of Science and Technology, Kowloon, China

**Keywords:** aggregation-induced emission, fluorescence, ELISA, drug residues, foodstuffs analysis

## Abstract

A new fluorescent “turn-on” probe-based immunosensor for detecting drug residues in foodstuffs was established by combining the mechanism of aggregation-induced emission (AIE) and an indirect competitive enzyme-linked immunosorbent assay (ELISA). In this study, a luminogen, with negligible fluorescence emission (TPE-HPro), aggregated in the presence of H_2_O_2_, and exhibited astrong yellow emission based on its AIE characteristics. This AIE process was further configured into an immunoassay for analyzing drug residues in foodstuffs. In this approach, glucose oxidase (GOx) was used as an enzyme label for the immunoassay and triggered GOx/glucose-mediated H_2_O_2_ generation, which caused oxidation of TPE-HPro and a “turn-on” fluorescence response at 540 nm. To quantitatively analyze the drug residues in foodstuffs, we used amantadine (AMD) as an assay model. By combining the AIE-active “turn-on” fluorescent signal generation mechanism with conventional ELISAs, quantifying AMD concentrations in chicken muscle samples was realized with an IC_50_ (50% inhibitory concentration) value of 0.38 ng/mL in buffer and a limited detection of 0.06 μg/kg in chicken samples. Overall, the conceptual integration of AIE with ELISA represents a potent and sensitive strategy that broadens the applicability of the AIE-based fluorometric assays.

## Introduction

The enzyme-linked immunosorbent assay (ELISA) is an extensively used immunoassay to detect the concentration of protein biomarkers and small molecules for clinical diagnosis, environmental monitoring, and food analysis (Suri et al., 2009; Chikkaveeraiah et al., 2012; Cheng et al., 2017; Wang et al., 2017). In conventional ELISAs, the enzyme-conjugated antibody simultaneously uses its specific immune recognition and bio-catalytic capabilities (Clark et al., 1986). Although conventional ELISAs are simple to use, effective, and commercially available, they suffer from moderate sensitivity, and are therefore unsuitable for analyzing low concentration analytes (Zhang et al., 2015b; Wang et al., 2016; Chen et al., 2019). A variety of fluorogenic ELISAs use fluorescent molecules or nanoparticles and have attracted increasing attention due to their higher sensitivity compared with traditional ELISAs (Li et al., 2015a; Fu et al., 2017; Sharma et al., 2018). Current fluorescent immunoassays have focused on the extensive synthesis of antibody and fluorescent molecules/nanomaterials conjugates and/or design of fluorescent signal mechanisms instead of enzymatic antibody labeling, as in traditional ELISA (Liu et al., 2013; Hlavácek et al., 2016; Sun et al., 2016). However, these fluorogenic ELISAs still have several limitations: (I) traditional organic fluorophores are vulnerable to photobleaching; (II) the synthesis of fluorescent nanoparticles is complicated and time-consuming; (III) the bio-conjugation process affects the stability of fluorescence probes and antibody activity; and (IV) aggregation-induced quenching results in fluorescent intensity decay. To solve these critical issues, fluorogens with aggregation-induced emission (AIE) properties might be a useful alternative.

Recently, fluorogens with AIE characteristics have emerged as a new class of fluorescent materials to detect various analytes (Hong et al., 2009; Kwok et al., 2015; Mei et al., 2015; Gao and Tang, 2017; Xia et al., 2018). AIE luminogens (AIEgens) are non-emissive in their molecularly dissolved state but exhibit strong emission in their aggregated state, the opposite of aggregation-caused quenching (ACQ), which is observed in conventional fluorophores (Luo and Xie, 2001; Zhang et al., 2014; Cai et al., 2018; He et al., 2018). Due to the unique AIE-based fluorogenic process, AIEgens have become a versatile and potent strategy for designing various sensors such as for gas (Zhang et al., 2015a), metal ions (Feng et al., 2018), and pH changes (Zhao et al., 2016). Until recently, few groups have designed new approaches that integrate immunoassays and fluorogens with AIE (Wang et al., 2014; Engels et al., 2016; Xiong et al., 2018). However, a hurdle for practical applications is their incompatibility with conventional immunoassay platforms, because they additionally require complicated chemical reactions to activate the AIE process, such as synthesis and the modification of AIE nanoparticles (Li et al., 2015b; Zhang et al., 2018) and a Cu^+^-catalyzed click reaction to form AIE polymers (Yuan et al., 2017). Therefore, the major challenge in developing an AIE-based immunosensor is designing a straightforward fluorescent signal generation methodology that is compatible with the current ELISA platform, which can be directly applicable with AIE-triggered fluorescent “turn-on.”

In this work, we designed a fluorogenic ELISA based on an AIE-active “turn-on” fluorescent probe, which can be directly applied to current immunoassay platforms and offers a much higher sensitivity. Previous studies in our group suggested that the AIE-active bioprobe, TPE-HPro, can sense hydrogen peroxide, which can be further used for a sensitive glucose detection in serum samples (Song et al., 2016b). Based on this principle, we developed a glucose oxidase (GOx)-triggered fluorescent “turn-on” system that uses GOx-catalyzed glucose oxidation and TPE-HPro aggregation. In this strategy, GOx was used as an enzyme label in the immunosensor for H_2_O_2_ generation, which triggered TPE-HPro oxidation and enabled fluorescent “turn-on” detection. This straightforward approach is fully compatible with the current immunoassay platform and can be generally applicable to the detection of drug residues in foodstuffs, which broadens the applicability of the AIE-based signal transduction system.

## Materials and Methods

### Reagents and Instruments

D-glucose, hydrogen peroxide, acetic acid, and acetonitrile were purchased from Sigma-Aldrich (St. Louis, USA). AMD was purchased from Tokyo Chemical Industry Co., Ltd (Tokyo, Japan). Bovine serum albumin (BSA) was purchased from Amresco Inc. (Solon, USA). Glucose oxidase-labeled goat anti-mouse IgG (gtAm-GOx) was purchased from Abcam (Cambridge, UK), and horseradish peroxidase-labeled goat anti-mouse IgG (gtAm-HRP) was purchased from Jackson ImmunoResearch (West Grove, USA). Phosphate-buffered saline (PBS) and Tris-HCl were purchased from Solaribo (Beijing, China). TMB substrate was purchased from Beyotime Biotechnology (Shanghai, China). TPE-HPro was produced in our laboratory as previously described (Song et al., 2016b). The coating antigen AMD-OVA (amantadine-ovalbumin conjugates) and monoclonal antibodies against AMD (mAb 3F2) were produced in our laboratory as described (Wang et al., 2018). All other chemical reagents required for the experiments were of analytical grade and obtained from Sigma-Aldrich (St. Louis, USA). UV-Vis and fluorescence (FL) spectra were measured on a SpectraMax M5 microplate reader (Molecular Devices San Jose, CA, USA).

### AIE-Based Hydrogen Peroxide and GtAm-GOx Sensing

The cleavage of the phenyl boronic ester occurs only in high pH environment. In our previous work, we evaluated and optimized the pH effect on the AIE process (Song et al., 2016b). At pH >10, the cleavage of phenyl boronic ester occurs and shows a significant fluorescence emission. Furthermore, the fluorescence intensity increases weakly after a 30 min incubation with TPE-HPro and H_2_O_2_, which indicates the saturation of the cleavage reaction. Thus, for AIE-based H_2_O_2_ sensing, 120 μL of different H_2_O_2_ concentrations (0–60 μM) were mixed with 42 μL Tris-HCl buffer (38 mM, pH = 11.5). Then the mixtures were added into the TPE-HPro probe solutions (400 μM in acetonitrile) at a 9:1 volume ratio. The solutions were kept to react at 37°C for 30 min. The fluorescence spectra of the solutions were recorded using a microplate reader.

For AIE-based gtAm-GOx sensing, gtAm-GOx was serially diluted with deionized water 0–14 μg/mL. Then, glucose (50 mM in H_2_O) was mixed with the gtAm-GOx solutions at a 3:1 volume ratio and incubated at 37°C for 30 min. Subsequently, 120 μL of these solutions were mixed with 42 μL Tris-HCl buffer (38 mM, pH = 11.5). Finally, 135 μL of the mixture was incubated with 15 μL TPE-HPro probe solutions (400 μM in acetonitrile). Fluorescence intensity was measured after incubation at 37°C for 30 min.

### Food Sample Pretreatment

Negative chicken muscle samples were provided and confirmed by the National Veterinary Drug Safety Evaluation Center (Beijing, China). Samples were analyzed by the AIE-based and conventional methods after the following pretreatment: 1.00 g of the chicken muscle sample was exactly weighed and spiked with AMD at 0.5, 1.0, and 2.0 μg/kg. Then, 5 mL 1% acetic acid in acetonitrile was added and well homogenized for 2 min. Subsequently, the homogenate was centrifuged for 5 min at 4,000 g. Finally, 3 mL of the supernatant was evaporated under nitrogen to dryness at 40°C, and the residue was dissolved in PBS and analyzed using an indirect competitive ELISA (Wang et al., 2018).

### Fluorometric “Turn-On” Immunoassay for the Model Analyte

For the AIE-based fluorometric immunoassays, 100 μL AMD-OVA conjugates were added to a 96-well plate and incubated at 4°C overnight. The plate was then blocked with 150 μL 1% BSA in PBS at 37°C for 1 h. The plates were washed three times with washing buffer (PBS containing 0.025% Tween-20) then 50 μL mAb 3F2 was added with 50 μL AMD of varying concentration or samples for competitive reactions. After incubation at 37°C for 30 min, the plate was washed four times and 100 μL gtAm-GOx in PBS (containing 1% BSA and 0.01% Tween-20) was added and incubated for 30 min at 37°C. The plate was washed three times with washing buffer and three times with deionized water. Then, we added 120 μL glucose (50 mM in H_2_O) and incubated it for 30 min at 37°C. Finally, 42 μL Tris-HCl buffer (38 mM, pH = 11.5) was added and the mixtures were added into the TPE-HPro probe solutions (400 μM in acetonitrile) at a 9:1 volume ratio. FL intensity was recorded by the plate reader after incubation at 37°C for 30 min. The calibration curve was analyzed by a four-parameter logistic equation using OriginPro 9.1 (OriginLab, Northampton, MA, USA).

## Results and Discussion

### The TPE-HPro-Based “Turn-On” Sensor for H_2_O_2_ and GtAm-GOx

**Figure 1:**
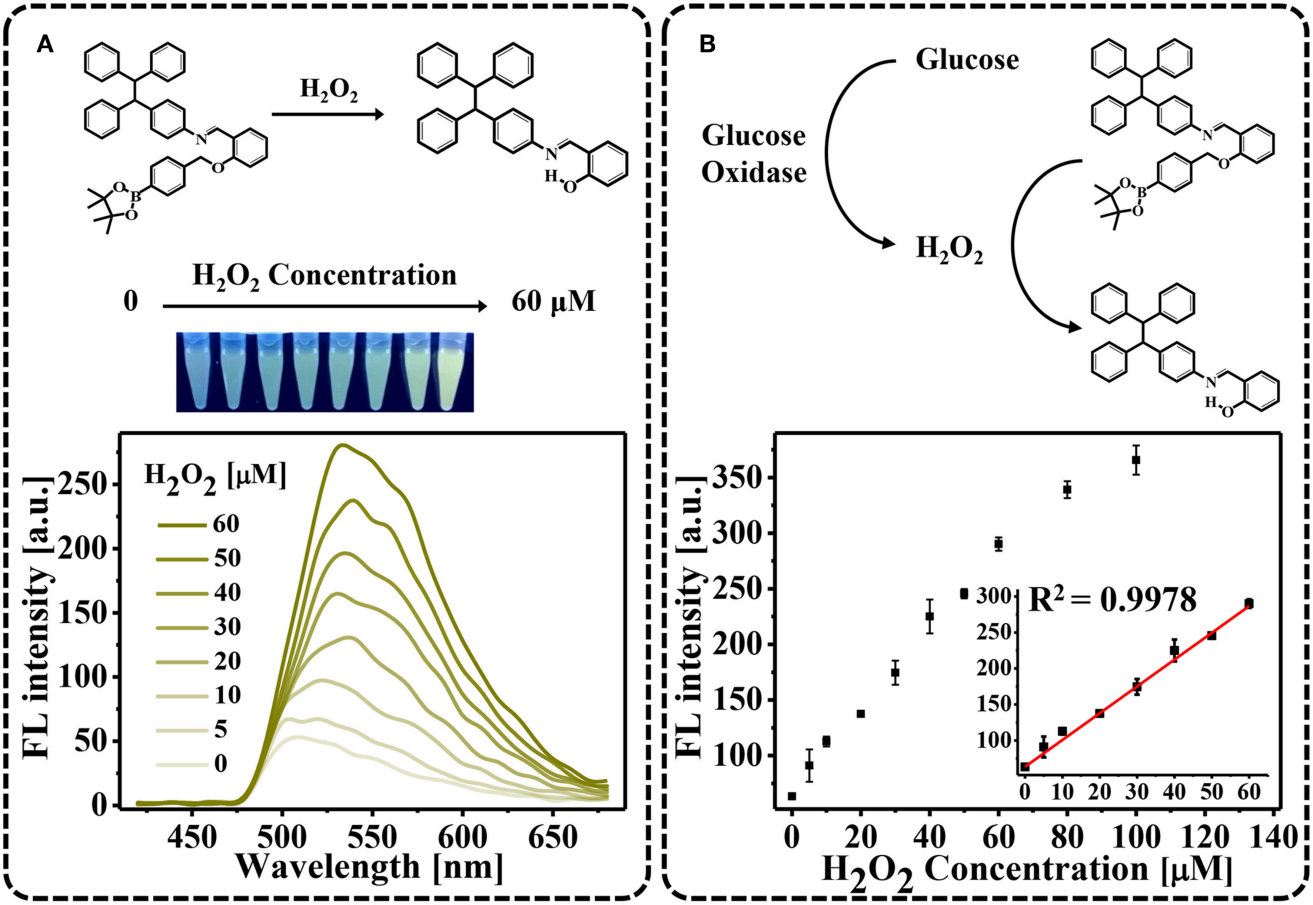
H_2_O_2_-triggred “turn-on” fluorescence. **(A)** Fluorescence spectra in the presence of varying H_2_O_2_ concentrations (0–60 μM). Photograph showing color changes of the solutions using a hand-held UV lamp; **(B)** Fluorescent intensities plotted against H_2_O_2_ concentration.

As a proof of principle, we first demonstrated that H_2_O_2_ oxidation of TPE-HPro to TPE-HPro-Ox could be used to sense H_2_O_2_. TPE-HPro, which consists of a TPE core structure and a phenyl boronic ester, exhibited negligible emission. In the chemical structure of TPE-HPro, the imine group acts as an emission mediator and can block the fluorescence emission by photo-induced electron transfer (PET) and a *cis-trans* isomerization process of C = N (Huxley et al., 2014; Song et al., 2016a). Therefore, in the presence of H_2_O_2_, the phenyl boronic ester is oxidized and cleaved. Due to the resulting intramolecular hydrogen bonding, the imine is conformed followed by a significant emission (Song et al., 2016b). The color changing of the AIE process could be clearly distinguished by the naked eye under a hand-held UV lamp and quantitatively measured by monitoring the FL spectra (Figure 1A). Different H_2_O_2_ concentration were used to oxidize TPE-HPro, and fluorescence “turn-on” was measured. Due to the AIE process, FL spectra showed an obvious characteristic peak at 540 nm, and the peak intensity at 540 nm gradually increased with H_2_O_2_ concentration from 0 to 60 μM. By plotting the FL intensity with H_2_O_2_ concentration (Figure 1B), the calibration curve with *R*^2^ = 0.9978 indicated a good linear response (Figure 1B inset). Moreover, the lowest H_2_O_2_ concentration (5 μM) can even be sensed with the fluorescence spectrophotometer. The sensitivity suggested that H_2_O_2_-triggered “turn-on” fluorescence could be further implemented to design a highly sensitive AIE-active “turn-on” sensor.

**Figure 2:**
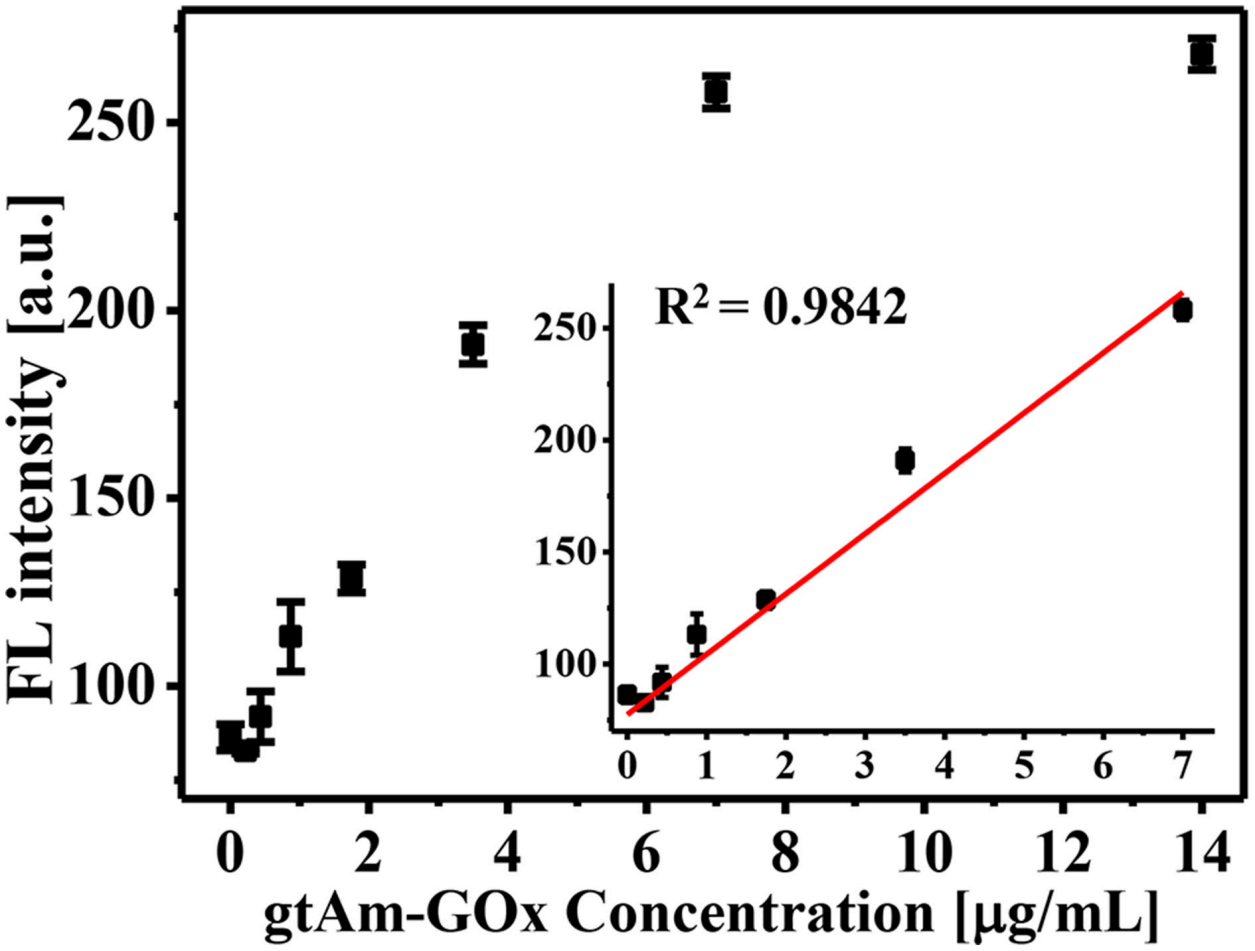
Glucose oxidase-mediated fluorescence “turn-on” using varying GOx-labeled secondary antibody concentrations (0–14 μg/mL).

When developing an enzyme-mediated immunoassay, the main challenge is finding a H_2_O_2_-generating enzyme that can modulate the AIE process for fluorescent measurements. To achieve this goal, we employed commercially available gtAm-GOx to effectively generate H_2_O_2_, as in our previous studies (Yu et al., 2017, 2018). As the concentration of the GOx-labeled secondary antibody increased, more H_2_O_2_ was generated by the enzymatic oxidation of glucose, which can be used to trigger the AIE process. We tested the sensitivity of GOx-catalyzed glucose oxidation for secondary antibody sensing. Final concentrations of GOx-labeled secondary antibody ranging from 0.2 to 14.0 μg/mL were investigated on AIE process (Figure 2). The FL intensity at 540 nm increased linearly with GOx-labeled secondary antibody concentrations in the range of 0–7.0 μg/mL (*R*^2^ = 0.9842; Figure 2 inset). As the concentration of gtAm-GOx increases, more H_2_O_2_ can be generated by the GOx/glucose enzymatic reaction, which leads to an increased level of the oxidation-stimulated AIE-process and fluorescence “Turn-On.” In this established enzyme-catalyzed signal transduction and oxidation reaction-triggered fluorescent “turn-on” sensor: (I) H_2_O_2_ was efficiently formed by the GOx-catalyzed oxidation reaction; (II) the AIEgen could be hydrolyzed by H_2_O_2_ even at 5 μM to produce strongly emissive AIE aggregates. Therefore, we expect to achieve highly sensitive detection by combining the AIE-active “turn-on” fluorescent sensor with conventional immunoassay technology.

### An AIE-Active “Turn-On” Fluorescent Probe-Based Indirect Competitive ELISA

**Figure 3:**
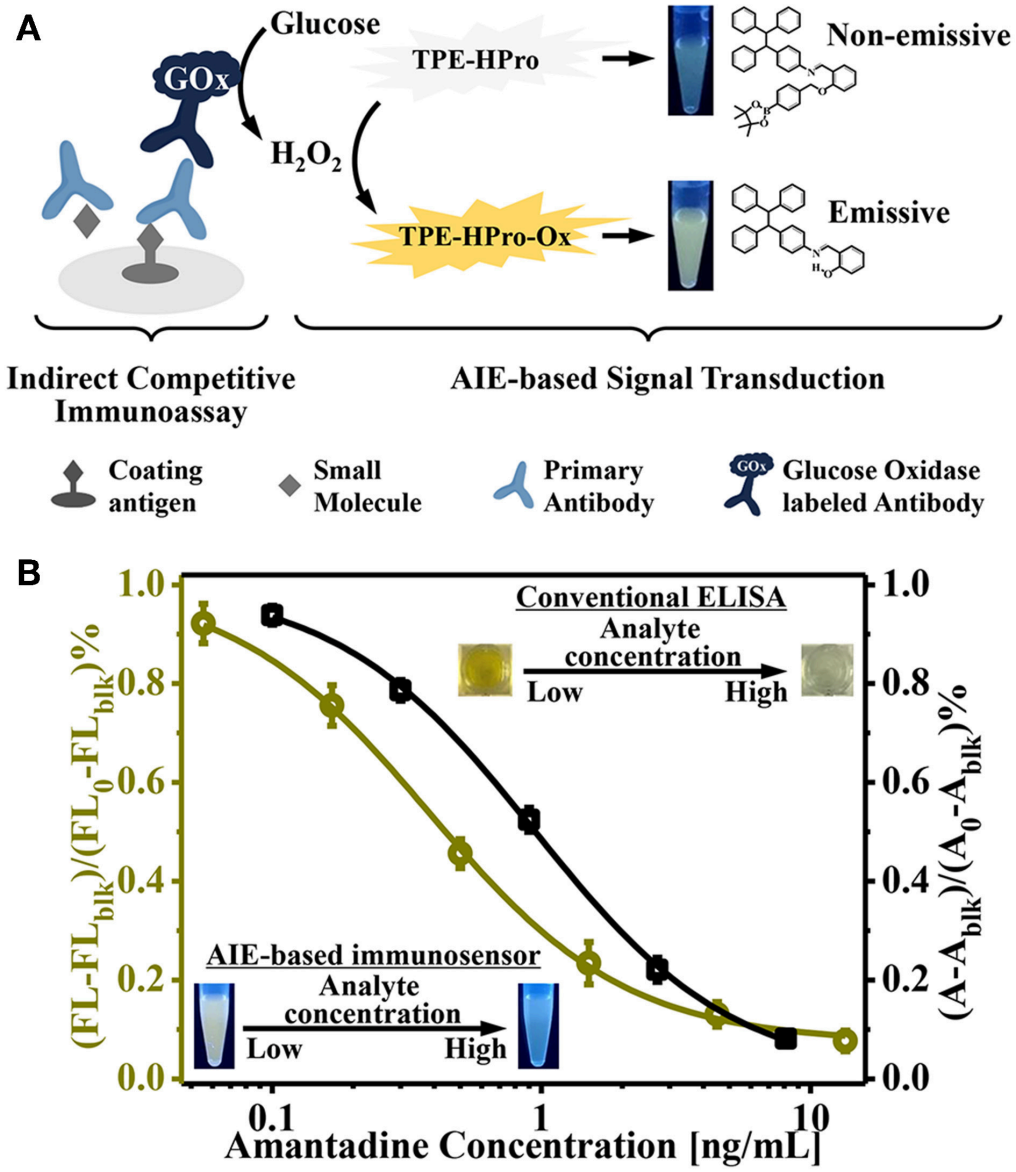
**(A)** Schematic illustration of the aggregation-induced emission (AIE)-active “turn-on” fluorescent immunosensor for indirect competitive ELISA; **(B)** Inhibition curve for quantitative determination of amantadine (AMD) by the newly designed AIE-based fluorescent “turn-on” immunosensor (left) and the conventional TMB-based method (right).

We further employed this AIE-active “turn-on” fluorescent sensor for highly sensitive indirect competitive ELISAs (Figure 3A). AMD-OVA conjugates were used as the coating antigen and were immobilized on a 96-well plate. For the competitive reaction, coated AMD-OVA conjugates competed with AMD in the buffer or sample for primary antibody (3F2) binding. Then, commercial GOx-labeled anti-mouse IgG antibodies were used as the secondary antibody for H_2_O_2_ formation and subsequent of AIE induction and fluorescent “turn-on” signal generation. Compared with traditional ELISAs, the only difference was that glucose rather than TMB was introduced into the signal generation step. Therefore, the procedures of our newly established AIE-based fluorescence “turn-on” immunoassay were nearly the same, and thus were fully compatible and comparable with current ELISA platforms. Instead of the absorbance values of a traditional ELISA, the fluorescence “turn-on” from the AIE-based sensor further amplified the signal output and enhanced the detection sensitivity.

To evaluate the sensitivity of our AIE-based indirect competitive ELISA, various concentrations of AMD were tested (0.06–13.5 ng/mL). Because of the competitive nature of this immunoassay platform, an increased AMD concentration led to intense competition, such that less primary and GOx-labeled secondary antibodies were bound on the ELISA plate; and subsequently less H_2_O_2_ was generated. Thus, no noticeable H_2_O_2_-induced “turn-on” fluorescence was obtained in the presence of high concentration of AMD. With decreasing AMD concentration of, fluorescence intensity gradually increased (Figure 3B). Relative fluorescence intensity [(FL-FL_blk_)/(FL_0_-FL_blk_)%] was used to quantitatively evaluate AIE process level (FL, FL_blk_, and FL_0_ were fluorescence intensities in the presence of varying AMD concentration, in the absence of secondary antibody, and at 0 ng/mL AMD, respectively).

We next compared our newly designed AIE-based ELISA with a conventional ELISA (Figure 3B). These results showed that the IC_50_ value of a conventional TMB/HRP-based ELISA was 0.93 ng/mL, while our newly established AIE-based fluorescence “turn-on” immunoassay provided a higher sensitivity; the IC_50_ value was 0.38 ng/mL. This suggests approximately 2.5-fold improvement in sensitivity. The limit of detection (signal-to-noise ratio of 3) was determined to be 0.06 ng/mL. This improved sensitivity relied on the following two principles: first, H_2_O_2_ was efficiently generated by the enzyme-catalyzed oxidation reaction; and second, the production of strong emissive AIE aggregates could be triggered by low concentrations of H_2_O_2_. These unique features make this “turn-on” fluorescent immunosensor an attractive immunoassay platform for highly sensitive detection.

### Applying the “Turn-On” Fluorescent Immunoassay to Detecting Drug Residues in Foodstuffs

**Figure 4:**
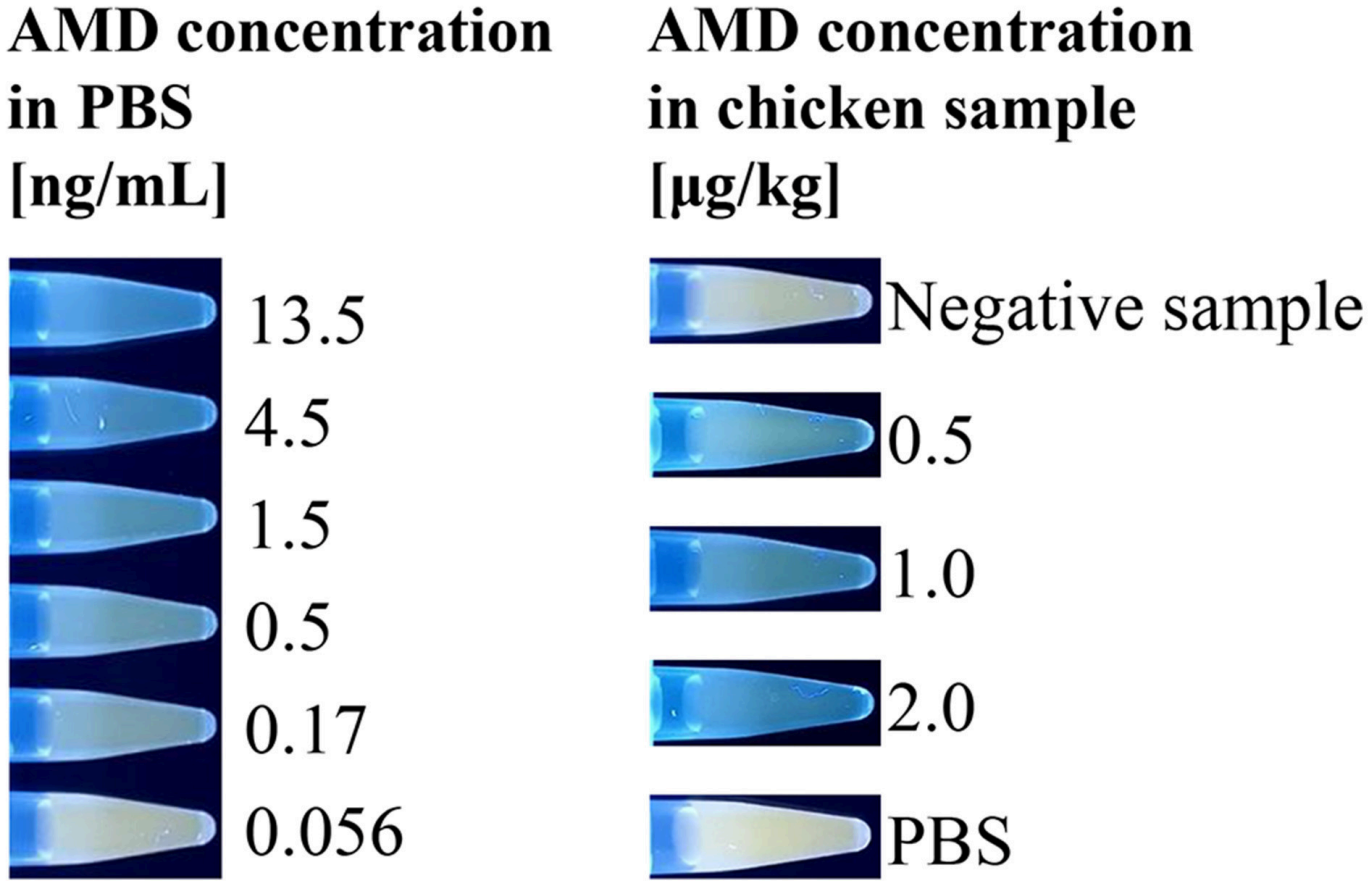
Emission photos of the AIE-based indirect competitive immunoassay using a hand-held UV lamp. Positive food samples containing AMD compete with the coated AMD-OVA on the ELISA plate, inhibiting the binding of GOx-labeled antibody, which catalyzes the oxidation reaction that stimulates fluorescent signal generation.

Finally, having successfully shown that the assay could quantify AMD, the “turn-on” fluorescent immunosensor was further evaluated using real food samples. AMD-contaminated chicken samples were used as a model. Chicken muscle samples were spiked with AMD at concentrations of 0.5, 1.0, and 2.0 μg/kg. In the indirect competitive immunoassay, spiked AMD in chicken samples competed with coating antigen for binding of the primary antibody. Thus, as the level of competition increased, less H_2_O_2_ was generated by GOx, which decreased the AIE-based fluorescence “turn-on.” Because of the complexity of chicken muscle samples, a pretreatment step was required. The color intensity change obtained for negative and positive chicken samples could be visually distinguished using a hand-held UV lamp (Figure 4). To quantitatively analyze AMD concentration in chicken muscle samples, fluorescence intensities were recorded by a microplate reader. As shown in Table 1, the recovery rates ranged from 88.2 to 91.7% with CVs of 8.3 to 11.8%. These results were comparable to our previous study based on conventional ELISAs for AMD detection, which used the same primary antibody (Wang et al., 2018). Therefore, our newly designed AIE-based fluorescent “turn-on” indirect competitive immunoassay was capable of detecting AMD in complex food matrices with high sensitivity.

**Table 1:** AMD detection rates from spiked chicken muscle samples at different concentrations (*n* = 5).

**Added**	**Found**		
**Concentration [μk/kg]**	**Mean [μg/kg]**	**Recovery [%]**	**CV [%]**
0.5	0.44	88.2	11.8
1.0	0.89	89.3	9.1
2.0	1.83	91.7	8.3

## Conclusion

In conclusion, we developed an AIE-based fluorescence “turn-on” immunoassay by combining a GOx-triggered AIE process with an indirect competitive ELISA for the highly sensitive detection of drug residues in foodstuffs. In this approach, the fluorescence “turn-on” is triggered by the GOx-catalyzed oxidation of glucose and H_2_O_2_-stimulated TPE-HPro aggregation. This cascade reaction makes use of the catalytic ability of the enzyme and the AIE characteristics, which lead to the signal amplification of the immunoassay. The AIE-based fluorogenic ELISA has much higher sensitivity due to the efficient generation of H_2_O_2_, which leads to a high fluorescence emission at low concentrations of H_2_O_2_. Compared with conventional HRP/TMB-based ELISAs, this newly designed immunoassay offers a 2.5-fold enhancement in sensitivity. Furthermore, this novel sensing strategy can be directly adapted to the current indirect competitive conventional ELISA format using an H_2_O_2_-triggered AIE fluorescence “turn-on” as the signal generation mechanism. This represents an alternative to the existing commercial immunoassay. More importantly, this approach can be applied to quantifying AMD in real food samples. Therefore, the developed AIE-based fluorescence “turn-on” ELISA has excellent potential for use in the testing of other drug residues in food samples.

## Author Contributions

WY planned and supervised the experiments, processed the raw data, and wrote the manuscript. YL synthesized TPE-HPro. BX performed the experiments. MM, CC, CL, and XY discussed the results and processed the data. ZW, KW, BT, and JS revised and approved the manuscript. All authors reviewed the manuscript and approved it for submission.

### Conflict of Interest Statement

The authors declare that the research was conducted in the absence of any commercial or financial relationships that could be construed as a potential conflict of interest.
